# Evaluation of a Whole-Liver Dixon-Based MRI Approach for Quantification of Liver Fat in Patients with Type 2 Diabetes Treated with Two Isocaloric Different Diets

**DOI:** 10.3390/diagnostics12020514

**Published:** 2022-02-16

**Authors:** Valentina Brancato, Giuseppe Della Pepa, Lutgarda Bozzetto, Marilena Vitale, Giovanni Annuzzi, Luca Basso, Carlo Cavaliere, Marco Salvatore, Angela Albarosa Rivellese, Serena Monti

**Affiliations:** 1 IRCCS Synlab SDN, 80143 Naples, Italy; luca.basso1591@gmail.com (L.B.); carlo.cavaliere@synlab.it (C.C.); direzionescientifica.irccssdn@synlab.it (M.S.); 2 Department of Clinical Medicine and Surgery, University of Naples Federico II, 80131 Naples, Italy; gdp0206@libero.it (G.D.P.); lutgarda48@gmail.com (L.B.); marilena.vitale@yahoo.it (M.V.); annuzzi@unina.it (G.A.); rivelles@unina.it (A.A.R.); 3 Institute of Biostructures and Bioimaging, National Research Council, 80145 Naples, Italy; serena.monti@ibb.cnr.it

**Keywords:** type 2 diabetes, hepatic fat fraction, Dixon MRI, magnetic resonance spectroscopy

## Abstract

Dixon-based methods for the detection of fatty liver have the advantage of being non-invasive, easy to perform and analyze, and to provide a whole-liver coverage during the acquisition. The aim of the study was to assess the feasibility of a whole-liver Dixon-based approach for liver fat quantification in type 2 diabetes (T2D) patients who underwent two different isocaloric dietary treatments: a diet rich in monosaturated fatty acids (MUFA) and a multifactorial diet. Thirty-nine T2D patients were randomly assigned to MUFA diet (*n* = 21) and multifactorial diet (*n* = 18). The mean values of the proton density fat fraction (PDFF) over the whole liver and over the ROI corresponding to that chosen for MRS were compared to MRS-PDFF using Spearman’s correlation (ρ). Before–after changes in percentage of liver volume corresponding to MRI-PDFF above thresholds associated with hepatic steatosis (LV%_TH_, with TH = 5.56%, 7.97% and 8.8%) were considered to assess the proposed approach and compared between diets using Wilcoxon rank-sum test. Statistical significance set at *p* < 0.05. A strong linear relationship was found between MRS-PDFF and MRI-PDFFs (ρ = 0.85, *p* < 0.0001). Changes in LV%_TH%_ were significantly higher (*p* < 0.05) in the multifactorial diet than in MUFA diet (25% vs. 9%, 35% vs. 12%, and 38% vs. 13% decrease, respectively, for TH = 5.56%, 7.97%, and 8.8%) and this was reproducible compared to results obtained using the standard liver fat analysis. A volumetric approach based on Dixon method could be an effective, non-invasive technique that could be used for the quantitative analysis of hepatic steatosis in T2D patients.

## 1. Introduction

Non-alcoholic fatty liver disease (NAFLD) refers to a condition encompassing multiple progressive liver disorders, ranging from simple hepatic steatosis, often called non-alcoholic fatty liver (NAFL), to nonalcoholic steatohepatitis (NASH). NAFLD and type 2 diabetes (T2D) are common conditions that regularly co-exist and can act synergistically to drive adverse outcomes [1,2]. Treatment options for NAFLD in patients with T2D are still a debated topic. In these patients, dietary treatments able to reduce hepatic fat content and improve liver histology are highly warranted. Open topics are also related to the techniques able to measure liver fat and detect fatty liver. The most reliable method for the detection of fatty liver is hepatic needle biopsy [3], which is currently the gold standard procedure in the diagnosis of fatty liver disease. However, a biopsy is an invasive technique, limited to the sample taken, and it may lead to misdiagnosis [4,5].

For larger-scale studies, different indexes based on aggregate scores from anthropometric and metabolic parameters has been proposed, such as the fatty liver index, the SteatoTest^®^ and the NAFLD liver fat score for the detection of NAFL, or NAFLD fibrosis score and fibrosis 4 calculator for the detection of liver fibrosis [6]. Although simple and non-invasive, the diagnostic performance of these indexes is difficult to compare, as they have been designed and validated against different standards. Furthermore, performances of FIB-4 and NAFLD fibrosis score to rule in advanced fibrosis are rather inadequate [6].

This led to an urgent need to discover novel alternatives for detection of fatty liver that are (i) non-invasive and (ii) cover the entire liver volume.

In recent years, magnetic resonance imaging (MRI), in particular the chemical-shift imaging (CSI) approach, and magnetic resonance spectroscopy (MRS) have become accurate methods to non-invasively quantify liver triglyceride concentration based on the difference in the resonant frequencies of water and fat [7]. Both methods are able to measure hepatic proton density fat fraction (PDFF), defined as the amount of protons bound to fat divided by the amount of all protons in the liver, including those bound to fat and water [8]. Single-voxel MRS estimates PDFF by directly measuring each water and fat peak, whereas CSI by MRI indirectly estimates PDFF using the signal interference between water and fat peaks. Therefore, MRS has been considered more accurate than MRI in measuring PDFF and represented the non-invasive gold standard in the quantification of hepatic steatosis due to its high sensitivity and dynamic range [8]. However, this technique has several limitations that prevent its widespread adoption for clinical and research applications. First, a skilled technologist is required to collect the data and specific analysis software to analyze those data. Then, MRS is limited in spatial coverage since it cannot cover the whole liver, involves a long acquisition time, and has high cost. Moreover, sampling errors are common due to the small sampling volumes considered [9]. Although these effects could be compensated by using data acquisition from multiple voxels, this solution has the disadvantage of an increased scan time [10]. On the other hand, MRI-based methods have the advantage of providing a full coverage of the liver during the acquisition, and of being easier to perform and analyze. To fully profit from the larger spatial coverage with MRI, several techniques exploiting the different physical and chemical properties of water and fat protons have been developed for hepatic fat quantification. Among the MRI methods to date, the most widely used is the chemical shift-based water–fat separation method proposed by Dixon, which is based on the different resonance frequencies between protons bound in a fat and water molecule [11]. One of the simplest implementations of the Dixon method is the so-called two-point Dixon method, in which images are acquired at specific echo times when water and fat signals are added and subtracted, producing “in-phase” and “opposed phase” images, respectively. In the fatty liver, there is signal loss in the opposed phase images and the liver appears dark. Although more complicated techniques based on the chemical shift effect have been developed to accurately define liver fat (e.g., multipoint Dixon) [12,13,14,15], this sequence is available on every scanner, is part of all upper abdominal MRI protocols, and is often used in clinical applications due to its simplicity [16]. Although the multipoint Dixon tends to be less vulnerable to reconstruction errors, the two-point Dixon is more desirable in the clinical practice, such as in situations where the acquisition time is critical (e.g., dynamic imaging or breath-hold imaging) [17].

MRS has been found to demonstrate excellent diagnostic performance for liver applications and showed close correlation with histological results [18,19,20]. Then, several studies were performed using Dixon-based techniques to quantify liver fat content with MRS as reference standard and demonstrated equivalent accuracy to MRS and a strong correlation with MRS, as well as with histology, for different applications involving NAFLD patients [9,20,21,22]. 

Given the urgent need to discover non-invasive and whole-liver based approaches for liver fat quantification, and considering the higher prevalence of NAFLD in T2D patients [23], we aimed at evaluating a novel whole-liver Dixon-based approach for quantification of liver fat. Preliminarily, a comparison of the Dixon method with the reference standard MRS and a standard analysis of liver fat were performed to assess the reproducibility of PDFF using both the traditional MRS and the Dixon method and validate the proposed approach. The latter was based on the quantification of percentage of liver volume with PDFF above thresholds indicating hepatic steatosis and, other than having the advantages pertaining to MRI-based methods, it allowed assessment of the whole-liver coverage, providing more information compared to the mere average of PDFF over the whole liver. We tested the approach in patients with T2D treated with two different isocaloric dietary approaches: a diet rich in monosaturated fatty acids (MUFA), already shown to be effective in reducing liver fat in patients with T2D [24], and a multifactorial diet rich in fiber, MUFA, n-6 and n-3 polyunsaturated fatty acids, polyphenols, and vitamins D, E, and C that has been shown to induce a more pronounced reduction in liver fat than MUFA diet [25].

## 2. Materials and Methods

### 2.1. Patient Characteristics and Study Design

The study protocol was reviewed and approved by the local Ethics Committee and was conducted in accordance with the ethical principles outlined in the Declaration of Helsinki and the Good Clinical Practice guidelines. All participants provided written informed consent before recruitment (number of the approval: 16/17). Eligible patients were men and women with T2D, overweight/obese, with high waist circumference, 35–75 years-old, and in satisfactory blood glucose control with diet or drugs not affecting liver fat content. From April 2017 to January 2019, forty-nine patients with T2D regularly attending the diabetes outpatient clinic of the Federico II University Teaching Hospital (Naples, Italy), were randomized to a monocentric, two-arm, open-label, controlled trial. Twenty-six participants were randomly assigned to an 8-week isocaloric intervention with a proven beneficial diet rich in monosaturated fatty acids (MUFA diet), and 23 participants to an 8-week isocaloric intervention with a multifactorial diet rich in fiber, MUFA, n-6 and n-3 polyunsaturated fatty acids, polyphenols, and vitamins D, E, and C. All measurement (anthropometric and metabolic parameters, and liver fat content) were taken after 12 h of overnight fasting at baseline and after 8 weeks of dietary intervention [25]. Thirty-nine patients (MUFA diet *n* = 21, multifactorial diet *n* = 18), for which MRI and MRS data were available, were included in this analysis (Table 1).

### 2.2. Liver Fat Content

#### 2.2.1. MRS

Measurements of liver fat content by MRS was performed on a 3T magnetic resonance scanner (dStream, Philips Healthcare, Eindhoven, The Netherlands) equipped with the dStream Torso coil, placed on the patient chest, and the dStream Posterior coil, allowing for abdominal imaging. In order to position the spectroscopy voxel, multiplanar localizing images covering the whole liver were preliminarily acquired. A 20 × 20 × 20 mm single voxel was placed in the right lobe, avoiding liver edges, large vessels, and large bile ducts. The spectroscopy was obtained using a point-resolved spectroscopy sequence with Echo Time (TE) = 35 ms, Repetition Time for minutes (TRmin) = 3000 ms, 16 signal averages, and 1024 data points over 2000 Hz spectral width. MRS scan was triggered at the end of expiration using a respiratory belt to minimize breathing artifacts. Spectral analysis was performed offline with LCModel software (http://s-provench-er.com (accessed on 31 March 2017)). This software fits in vivo metabolite spectra by means of model resonances acquired under comparable scanning conditions from multiple compounds in standard phantom solutions [26]. Values of water peak (signal of water, Sw) and the sum of lipid peaks at 1.3, 0.9, and 1.6 ppm (signal of fat, Sf) were considered for quantification of liver fat. Signal decay correction for the different T2 and T1 decay of water and fat was performed using mean T2 relaxation times of 30 and 52 ms and mean T1 relaxation time of 990 and 402 ms for water and fat, respectively [27]. Finally, PDFF was calculated as PDFF (%) = Sf/(Sf + Sw). The MRS-measured PDFF will be referred to as MRS-PDFF in the following paragraphs.

#### 2.2.2. MRI

A commercially available version of mDIXON sequence package was used to acquire fat and water images over the whole liver. The mDIXON technique combines a 2-point DIXON method with the implementation of flexible echo times. The following imaging parameters were analyzed: 3D T1-FFE sequence, 2-echoes: TE1 = 1.2 ms, TE2 = 2.3 ms, TR = 3.2 ms, Flip angle = 10, SENSE with acceleration factor 1.5 in phase-encoding direction, matrix = 264 × 218, field of view = 393 × 323 × 200 mm^3^, voxel size = 0.98 × 0.98 × 2.00 mm^3^.

**Figure 1:**
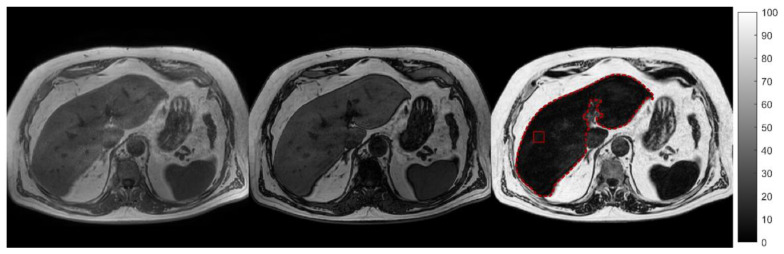
In-phase, opposed phase and MRI-PDFF map segmentation of the entire liver (dotted red line) with the corresponding ROI colocalized with that used for MRS (solid red line).

Data for each 2-echo mDIXON sequence were reconstructed on the MR system using the available standard single-peak spectral model of fat. Each sequence yielded four images per slice: water only, fat only, in-phase, and out-phase. A voxel-wise “signal fat-fraction” parameter map (MRI-PDFF map) was calculated voxel-by-voxel by means of an in-house software, using the previous equation, from the water and fat images automatically computed by the scanner. The whole liver was semi-automatically segmented in the MRI-PDFF maps by using the free tool ITK-snap (http://www.itksnap.org/pmwiki/pmwiki.php?n=Main.HomePage (accessed on 15 March 2021)). A cubic VOI with a side length of 20 mm was colocalized with the MRS voxel and placed on the MRI-PDFF map. Finally, MRI-PDFF values over both the VOI (MRI-PDFF_VOI_) and the whole liver segmentation (MRI-PDFF_WL_) were computed. Figure 1 shows an example of whole-liver segmentation and ROI placement in fat fraction maps.

### 2.3. Statistical Analysis

Data were expressed as means ± standard deviation. At first, an analysis aiming at comparing MRI-PDFFs (both VOI-based and whole liver-based) with the gold standard MRS-PDFF was performed to assess the reproducibility of PDFF using both the traditional MRS and the Dixon method and validate measurements used for the assessment of the proposed volumetric approach. In particular, Spearman’s correlation analysis was used to evaluate the correlation between MRI-PDFFs and MRS-PDFF, independently from the considered time-point and dietary treatment. Moreover, the inter-method agreement was assessed by Bland–Altman analysis. The same analysis was also performed to assess the correlation and agreement between MRI-PDFF_VOI_ and MRI-PDFF_WL_. Then, two types of dietary-based analyses were performed: the standard analysis, aiming at validating the proposed volumetric approach and for which the outcome was the reduction of liver fat, and the volumetric analysis, performed to assess the proposed approach, for which the outcome was the reduction in percentage of volume corresponding to liver fat content above a certain threshold. The statistical analysis was performed with MATLAB R2020a. Statistical significance was set at *p* < 0.05. Details on standard and volumetric analyses of liver fat will be provided in Section 2.3.1 and Section 2.3.2, respectively.

#### 2.3.1. Standard Analysis of Liver Fat

The outcome for standard analysis of liver fat was the reduction of liver fat after 8 weeks of dietary treatment. MRS-PDFF, MRI-PDFF_VOI_ and MRI-PDFF_WL_ at baseline and after 8 weeks were evaluated in all patients, both those assigned to the multifactorial diet and those assigned to the MUFA diet. Before–after intervention differences were assessed by using the paired Wilcoxon signed rank test. The effects of the two dietary interventions were tested by two-way repeated measures analysis of variance (ANOVA). Changes (8-week values−baseline values) and percentage changes ([8-week values−baseline values]/baseline values × 100) in liver fat content were also evaluated and compared between the two dietary treatments by using Wilcoxon rank-sum test.

#### 2.3.2. Volumetric Analysis of Liver Fat

The outcome for volumetric analysis performed to assess the proposed approach was the reduction in percentage of liver volume corresponding to liver fat content in the MRI-PDFF map that were above a certain threshold defining hepatic steatosis. For an easier reading, the notation LV%_TH_ will be used to indicate the percentage of liver volume (LV%) corresponding to liver fat content in the MRI-PDFF map that were above a certain threshold (TH) defining hepatic steatosis.

Volumetric analysis was firstly performed assuming MRI-PDFF value above 5.56% as a cut-off for steatosis [28]. 

Then, using MRS-PDFF as reference for hepatic steatosis assessment (namely assuming MRS-PDFF as label on the basis of 5.56% threshold), Receiver Operating Curve (ROC) analysis was performed to find the MRI-PDFF_VOI_ and MRI-PDFF_WL_ cut-off values that discriminated no steatosis from mild steatosis. The selected thresholds were obtained optimizing the Youden’s index and used to further test the volumetric approach.

Therefore, we tested the capability of three threshold values supposed to distinguish no steatosis from mild steatosis, to evaluate the percentage of liver volume with PDFF above that given threshold. The reduction in percentage of volume above the threshold was used to compare the two dietary treatments. A Wilcoxon rank-sum test was used to compare changes and percentage changes in LV%_TH_ between the two dietary treatments.

## 3. Results

### 3.1. Anthropometrics and Metabolic Parameters

As already reported in our previous paper [25], participants allocated to the two dietary intervention groups were comparable for the anthropometric and metabolic parameters at baseline. After the 8 weeks of dietary intervention, no significant differences in fasting plasma concentration of liver enzymes, glucose, insulin, triglycerides, total cholesterol, LDL and HDL cholesterol, and triglycerides were observed between baseline and end of the intervention, and between groups [25].

### 3.2. Correlation and Agreement of Liver Fat Content Measurements by MRI and MRS

Independently from the considered time point and the dietary treatment, a strong linear relationship was found between MRS-PDFF and MRI-PDFF (ρ = 0.85 when MRI-PDFF = MRI-PDFF_VOI_ and ρ = 0.85 when MRI-PDFF = MRI-PDFF_WL_, *p* < 0.0001), as well as between MRI-PDFF_VOI_ and MRI-PDFF_WL_ (ρ = 0.93, *p* < 5 × 10^−20^) (Figure S1a,c,e). Furthermore, a strong linear relationship was found between MRS-PDFF and MRI-PDFF changes, both as absolute (ρ = 0.7, when MRI-PDFF = MRI-PDFF_VOI_ and ρ = 0.78 when MRI-PDFF = MRI-PDFF_WL_, *p* < 0.0001), and percentage variation (ρ = 0.61 when MRI-PDFF = MRI-PDFF_VOI_ and ρ = 0.69 when MRI-PDFF = MRI-PDFF_WL_, *p* < 0.0001). Bland–Altman analysis revealed a systematic error in the Dixon based fat-signal fraction, which showed a prevailing overestimation of Dixon-based with respect to MRS with few outliers (mean difference -MD- = 2.07% and 95% confidence interval -CI- = [−2.93%, 7.1%] when MRI-PDFF was measured with ROI corresponding to the MRS voxel; MD = 2.52% and 95% CI = [−2.57%, 7.6%] when MRI-PDFF was measured with the entire liver). This could be also observed in the Bland–Altman analysis between MRI-PDFF_VOI_ and MRI-PDFF_WL_ (MD = −0.45; 95% CI = [−2.93%, 7.1%]) (Figure S1b,d,f).

### 3.3. Standard Analysis of Liver Fat

MRS-PDFF values significantly decreased after both the multifactorial diet (9.18 ± 7.78% vs. 5.22 ± 4.80%, *p* = 0.003) and the MUFA diet (9.47 ± 8.89% vs. 8.07 ± 8.52%, *p* < 0.05), and a significantly greater change was observed after the multifactorial diet (−4.0 ± 4.5%) than after the MUFA diet (−1.4 ± 2.7%) (*p* < 0.05). Percent reduction of MRS-PDFF was significantly greater with the multifactorial diet (−40 ± 33%) than the MUFA diet (−19 ± 25%) (*p* < 0.05) (Figure S2a,b). MRI-PDFF results confirmed these findings, showing a liver fat reduction after both the multifactorial diet and the MUFA diet, both considering the whole-liver analysis (10.6 ± 5.29% vs. 8.18 ± 4.31%, *p* = 0.003 in multifactorial diet; 11.7 ± 7.87% vs. 10.9 ± 7.93%, *p* = 0.015 in MUFA diet), and the VOI-based analysis (10.46 ± 7.03% vs. 6.96 ± 4.4%, *p* = 0.003 in multifactorial diet; 11.52 ± 9.15% vs. 10.7 ± 9.97%, *p* = 0.015 in MUFA diet). A significantly greater change was observed after the multifactorial diet than after the MUFA diet, both considering the whole-liver analysis (−2.5 ± 2.3% after the multifactorial diet vs. −0.8 ± 1.9% after the MUFA diet, *p* < 0.05), and the VOI based analysis (−3.5 ± 4.5% after the multifactorial diet vs. −0.8 ± 2.16% after the MUFA diet, *p* < 0.05). Percent reduction of MRI-PDFF_WL_ was significantly greater with the multifactorial (−22 ± 13.83%) than the MUFA diet (−7.12 ± 15.43%) (*p* = 0.007) (Figure S2d). The same was found considering MRI-PDFF_VOI_: −26.3 ± 20.52% in the multifactorial diet vs. −12.2 ± 19.93% in the MUFA diet (*p* = 0.030) (Figure S2f).

### 3.4. Volumetric Analysis of Liver Fat

**Figure 2:**
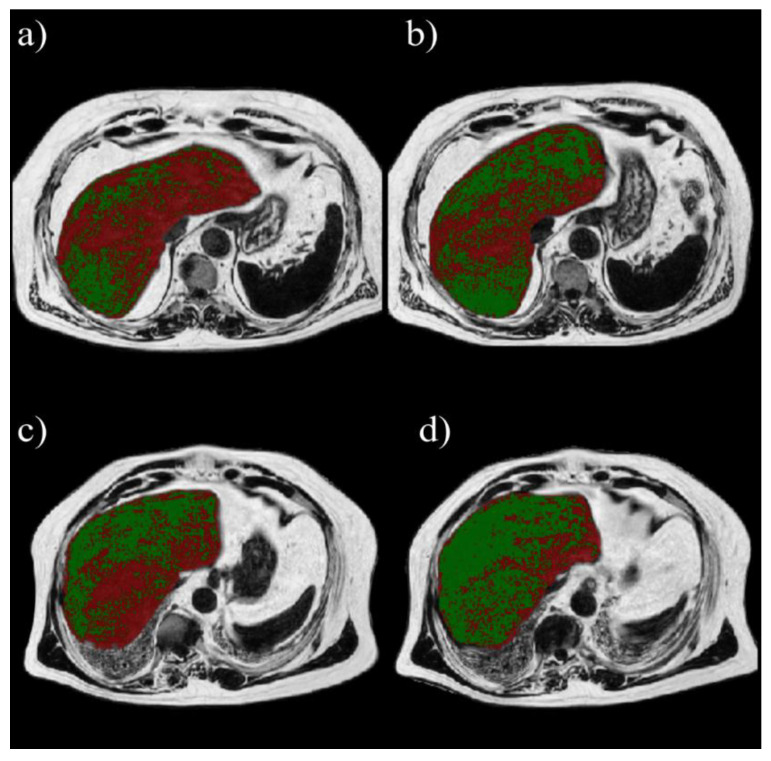
Examples of volumetric analysis. On the first row, a patient who underwent MUFA diet with percentage of liver volume with MRI-PDFF > 5.56% (red) decreasing post-treatment (**b**) with respect to pre-treatment (**a**) of 24.4%. On the second row, a patient who underwent multifactorial diet with percentage of liver volume with MRI-PDFF > 5.56% (red) decreasing post-treatment (**d**) with respect to pre-treatment (**c**) of 32.3%.

**Figure 3:**
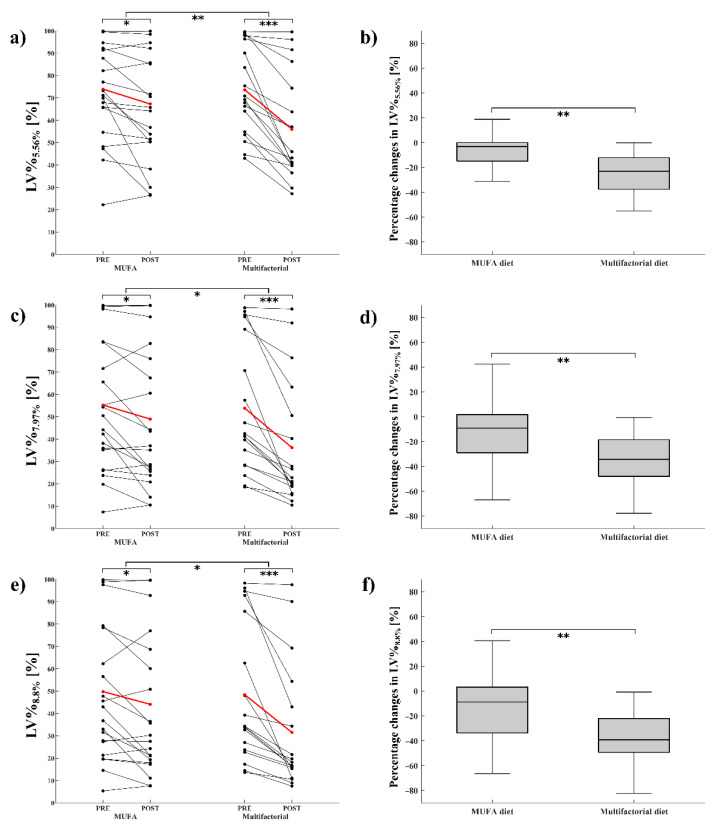
Absolute individual changes (left side) and percent changes (right side) in percentage of volume above thresholds for steatosis after the 8-week intervention with MUFA diet or multifactorial diet. Steatosis thresholds were set at 5.56% (**a**,**b**), 7.97% (**c**,**d**), and 8.8% (**e**,**f**). Asterisks indicates statistically significant differences (* = *p* < 0.05; ** = *p* < 0.005; *** = *p* < 0.0005). Abbreviations: MRI = Magnetic Resonance Imaging; PDFF = Proton Density Fat Fraction; VOI = Volume of Interest; and WL = Whole Liver.

Figure 2 shows an example of volumetric analysis in one patient. Assuming 5.56% as PDFF threshold, LV%_5.56_ decreased after both the MUFA diet (73.95 ± 21.95% at baseline, 67.3 ± 25.4% after 8 weeks, *p* = 0.01) and the multifactorial diet (73.64 ± 19.85% at baseline, 56.03 ± 23.65% after 8 weeks, *p* < 0.0001). A significantly greater change in LV%_5.56%_ was observed after the multifactorial diet (−17.61 ± 13.43%) than after the MUFA diet (−6.66 ± 11.06%) (*p* = 0.0027) (Figure 3a). The same happened for percentage changes (−9.3 ± 17.46% in MUFA vs. −25.17 ± 17.03% in multifactorial diet, *p* = 0.0032) (Figure 3b). 

With an area under the ROC curve (AUC) = 0.95, the optimal threshold for MRI-PDFF_VOI_ associated with the Youden index was 7.97%. Results obtained for 7.97% threshold were comparable to those for 5.56% threshold. In particular, LV%_7.97%_ decreased after both the MUFA diet (55.3 ± 29.5% at baseline, 49 ± 31.7% after 8 weeks, *p* = 0.02) and the multifactorial diet (53.8 ± 29.3% at baseline, 36.2 ± 27% after 8 weeks, *p* < 0.0001). A significantly greater change in LV%_7.97%_ (8-week minus baseline values) was observed after the multifactorial diet (−17.65 ± 15.5%) than after the MUFA diet (−6.27 ± 10.48%) (*p* = 0.01) (Figure 3c). The same happened for percentage changes (−12.17 ± 25.33% in MUFA diet versus −35.19 ± 20.69% in multifactorial diet, *p* = 0.0032) (Figure 3d). 

With an AUC = 0.97, the optimal threshold for MRI-PDFF_WL_ associated with the Youden index was 8.8%. Results obtained for 8.8% threshold were comparable to those for 5.56% and 7.97% thresholds. In particular, LV%_8.8%_ decreased after both the MUFA diet (50 ± 31% at baseline, 44 ± 32.7% after 8 weeks, *p* = 0.03) and multifactorial diet (48.3 ± 31.2% at baseline, 31.5 ± 28.1% after 8 weeks, *p* < 0.0001). A significantly greater change in LV%_8.8%_ was observed after the multifactorial diet (−16.9 ± 16.2%) than after the MUFA diet (−5.71 ± 10.14%) (*p* = 0.02) (Figure 3e). The same happened for percentage changes (−12.74 ± 27.12% in MUFA diet versus −37.47 ± 21.36% in multifactorial diet, *p* = 0.0046) (Figure 3f).

## 4. Discussion

In this study, we investigated the use of a volumetric approach based on Dixon method for quantification of liver fat in patients with T2D treated with two different isocaloric dietary approaches, namely an isocaloric multifactorial diet rich in fiber, MUFA, n-6 and n-3 polyunsaturated fatty acids, polyphenols, and vitamins D, E, and C, and an isocaloric diet rich in MUFA already shown to be effective in reducing liver fat in patients with T2D [24]. Based on the results already reported by Della Pepa et al. [25], the multifactorial diet could be considered the optimal dietary approach to prevent and treat NAFLD in patients with T2D with respect to MUFA diet. Specifically, although both diets were able to reduce liver fat content in patients with T2D, the impact was significantly greater with the multifactorial diet (40% decrease in liver fat content). The main outcome of the trial was the effect of the two dietary treatments on liver fat evaluated by MRS and, given the potential advantages of Dixon method for liver fat quantification, we applied a whole-liver Dixon-based approach for quantification and comparison of liver fat in a similar patient cohort. The proposed approach was based on the quantification of percentage of liver volume with liver fat content in the MRI-PDFF map that were above a certain threshold defining hepatic steatosis (LV%_TH_, with TH = 5.56%, 7.97% and 8.8%). Our results revealed that the proposed volumetric approach based on Dixon method could be an effective, non-invasive technique that can be used for the quantitative analysis of hepatic steatosis in patients with T2D treated with different dietary treatments, revealing reproducible results with respect to those obtained by using the standard liver fat analysis. In particular, percentage changes in LV%_TH%_ were greater in the multifactorial diet than in MUFA diet (25% vs. 9%, 35% vs. 12%, and 38% vs. 13% decrease, respectively, for TH = 5.56%, 7.97%, and 8.8%). The robustness of these findings was corroborated by reproducibility of liver fat measurements using both the traditional MRS and the Dixon method. The latter showed, considering the whole-liver coverage, a difference in the reduction of steatosis between the two dietary treatments that is comparable to the one observed in a single region of hepatic parenchyma as measured by MRS. This can prevent the occurrence of sampling biases possibly affecting results from volumetric analysis of liver fat.

Interestingly, the strong correlation found between MRS-PDFF and MRI-PDFF was in line with findings by a large number of previous studies investigating the accuracy of Dixon-based methods for liver fat estimation in different hepatic diseases, assuming MRS as reference standard [9,10,20,22]. 

To our knowledge, this is the first study aiming at investigating a MRI-based whole-liver approach for liver fat estimation that aims to go beyond both the standard MRS analysis of liver fat and the commonly used MRI approach for liver fat estimation based on average measurement of PDFF in regions of interest covering part of the liver or the whole-liver coverage [10,22,29,30].

Despite our encouraging results, our study suffers from several limitations. First, due to the small sample size, further studies on larger populations are needed to confirm the effectiveness of our approach. Furthermore, this study had no histologic evaluation, which is the gold standard of quantification of hepatic steatosis. Concerning the MR acquisition protocol, the limitation in our study is that we have only used basic techniques available to us, namely, a multiecho two-point Dixon (mDixon) technique for data acquisition and a standard single-peak spectral model of fat for image reconstruction. Since factors confounding the fat measurement (T1 and T2 bias, T2* decay, spectral complexity of fat, etc.), have not been addressed, our measurement represents the “signal fat-fraction”, and not a true “proton density fat-fraction” [31]. However, it should be considered that this sequence is available on every scanner, it is part of all upper abdominal MR protocols and is often used in clinical applications due to its simplicity, and this has a high impact in view of translation of our findings into clinical practice. Moreover, the mDixon technique with multiple echo times used in this study has been shown to improve fat-water separation, have better signal-to-ratio, and have the feasibility of breath hold [32].

Another issue to be considered is that there is still no exact standardized MRI fat fraction cutoff value for discriminating between normal and abnormal levels of liver fat. However, we determined hepatic steatosis at PDFF > 5.56%, which is a common threshold to distinguish normal liver from fatty liver. This threshold was derived from a large study by Szczepaniak et al. [28] and used in the majority of studies aiming at comparing MRS and MRI-PDFF and assuming MRS as reference standard for the assessment of hepatic steatosis [20,25,33,34,35]. Given the need for protocol-specific establishment of cutoffs for liver fat content, we also considered cut-off values obtained from ROC analysis of MRI-PDFF values that discriminated no steatosis from mild steatosis assuming MRS-PDFF as a reference in the quantification of hepatic steatosis. 

Previous studies investigated different thresholds for MRS and MRI-PDFF arising from their results based on histological findings [32,36]. Unfortunately, we could not estimate MRS and MRI thresholds based on histopathological results, due to missing histologic evaluation. Moreover, we opted for not evaluating other thresholds from literature than 5.56% by MRS. This is mainly because this value was derived from a large study. Moreover, other thresholds arising from MRS or MRI were derived from smaller studies or established based on MRI-PDFF methods with different settings and characteristics [10,22,36,37]. 

Finally, it is important to underline that our study has been performed in a specific population of patients with T2D treated with dietary interventions. However, similar results could be detected also in patients without T2D since the amount of fat in the liver is driven in particular by insulin resistance also in non-diabetic patient [38], although NAFLD has a higher incidence and severity in T2D [23]. Although the method was able to detect the effect of different type of diets, its capability in quantifying liver fat is independent from the patient types and their compliance to diets. Therefore, further studies evaluating the proposed whole-liver Dixon-based approach in patients without T2D, and not necessarily undergoing dietary treatments, should be considered.

Despite the above, the strength of our study is the confirmation of results obtained by standard liver fat analysis by using a method that has the advantage of being based on the Dixon method, which showed a high correlation with fat fraction estimated with MRS. Moreover, the proposed approach certainly presents the advantages derived from MRI-based methods (non-invasiveness, whole-liver coverage during the acquisition, and ease of execution and evaluation), but offered the added value of allowing the assessment of the whole-liver coverage providing a more complete information compared to what the mere average value of MRI-PDFF over the whole liver.

## 5. Conclusions

In conclusion, a volumetric approach based on Dixon MRI could be an effective, non-invasive technique for the quantitative analysis of hepatic steatosis in patients with T2D. However, its capability in quantifying liver fat independently from the patient types and their compliance to diets paves the way for further studies and research lines evaluating the proposed whole-liver Dixon-based approach in patients without T2D and not necessarily undergoing dietary treatments.

## Figures and Tables

**Table 1:** Baseline characteristics of the participants. Data are *n* (%) or mean (Standard Deviation). Not statistical differences between the two groups. Abbreviations: BMI = body mass index; HbA1c = glycated hemoglobin.

	MUFA-Diet (*n* = 21)	Multifactorial-Diet (*n* = 18)
Sex		
Male	12 (57%)	10 (56%)
Female	9 (43%)	8 (44%)
Age (years)	64 (5)	64 (6)
BMI (kg/m^2^)	31 (3)	32 (4)
HbA1c (%)	6.5 (0.6)	6.5 (0.4)
Diabetes therapy:		
Diet	5 (24%)	5 (28%)
Glucose lowering drugs	16 (76%)	13 (72%)
Other drugs:		
Statin	13 (62%)	9 (50%)
Anti-hypertensive	19 (90%)	16 (89%)
Anti-platelet	5 (24%)	6 (34%)

## Data Availability

Not applicable.

## Supplementary Materials

The following supporting information can be downloaded at: https://www.mdpi.com/article/10.3390/diagnostics12020514/s1: Figure S1: Linear correlation and Bland-Altman analyses of mean fat fraction measured by MRI and MRS. (a) correlation analysis and (b) Bland-Altman analysis of MRS-PDFF and MRI-PDFF in whole-liver; (c) correlation analysis and (d) Bland-Altman analysis of MRS-PDFF and MRI-PDFF in liver VOI; (e) correlation analysis and (f) Bland-Altman analysis of MRI-PDFF in liver VOI and MRI-PDFF in whole-liver. In Bland-Altman analysis, the thick solid line represents the mean value and dashed lines represent 95% confidence intervals. PDFF, proton density fat fraction; MRS, magnetic resonance spectroscopy; MRI = Magnetic Resonance Imaging; VOI = Volume Of Interest; WL = Whole Liver. Figure S2: Absolute individual changes (left side) and percent changes (right side) in liver fat content after the 8-week intervention with MUFA diet or multifactorial diet, measured with MRS (a,b) and Dixon-MRI, with whole-liver-based (c,d) or VOI-based analysis (e,f). Asterisks indicates statistically significant differences (* = *p* < 0.05; ** = *p* < 0.005; *** = *p* < 0.0005). Abbreviations: MRI = Magnetic Resonance Imagnig; PDFF = Proton Density Fat Fraction; VOI = Volume Of Interest; WL = Whole Liver.
