# Supplementary material for: Evaluation of a Whole-Liver Dixon-Based MRI Approach for Quantification of Liver Fat in Patients with Type 2 Diabetes Treated with Two Isocaloric Different Diets
Source: Diagnostics (Basel). 2022 Feb 16;12(2):514. doi: 10.3390/diagnostics12020514 (PMC8871286; doi:10.3390/diagnostics12020514)
Supplement: Supplementary file 1 [file diagnostics-12-00514-s001.zip › diagnostics-1564759-supplementary.pdf]

Supplementary Materials

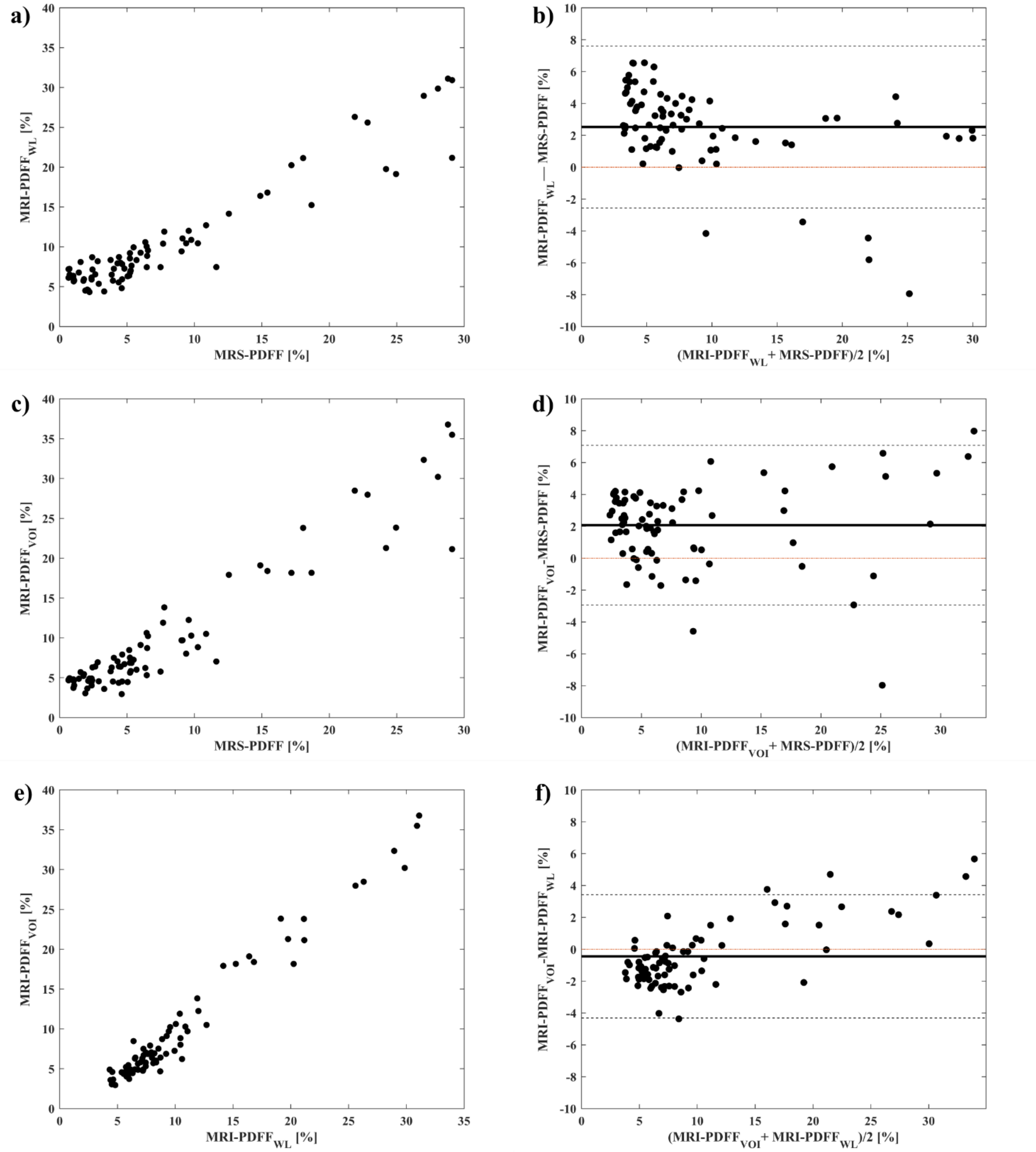

Figure S1. Linear correlation and Bland-Altman analyses of mean fat fraction measured by MRI and MRS. (a) correlation analysis and (b) Bland-Altman analysis of MRS-PDFF and MRI-PDFF in whole-liver; (c) correlation analysis and (d) Bland-Altman analysis of MRS-PDFF and MRI-PDFF in liver VOI; (e) correlation analysis and (f) Bland-Altman analysis of MRI-PDFF in liver VOI and MRI-PDFF in whole-liver. In Bland-Altman analysis, the thick solid line represents the mean value and dashed lines represent 95% confidence intervals. PDFF, proton density fat fraction; MRS, magnetic resonance spectroscopy; MRI = Magnetic Resonance Imaging; VOI = Volume Of Interest; WL = Whole Liver.

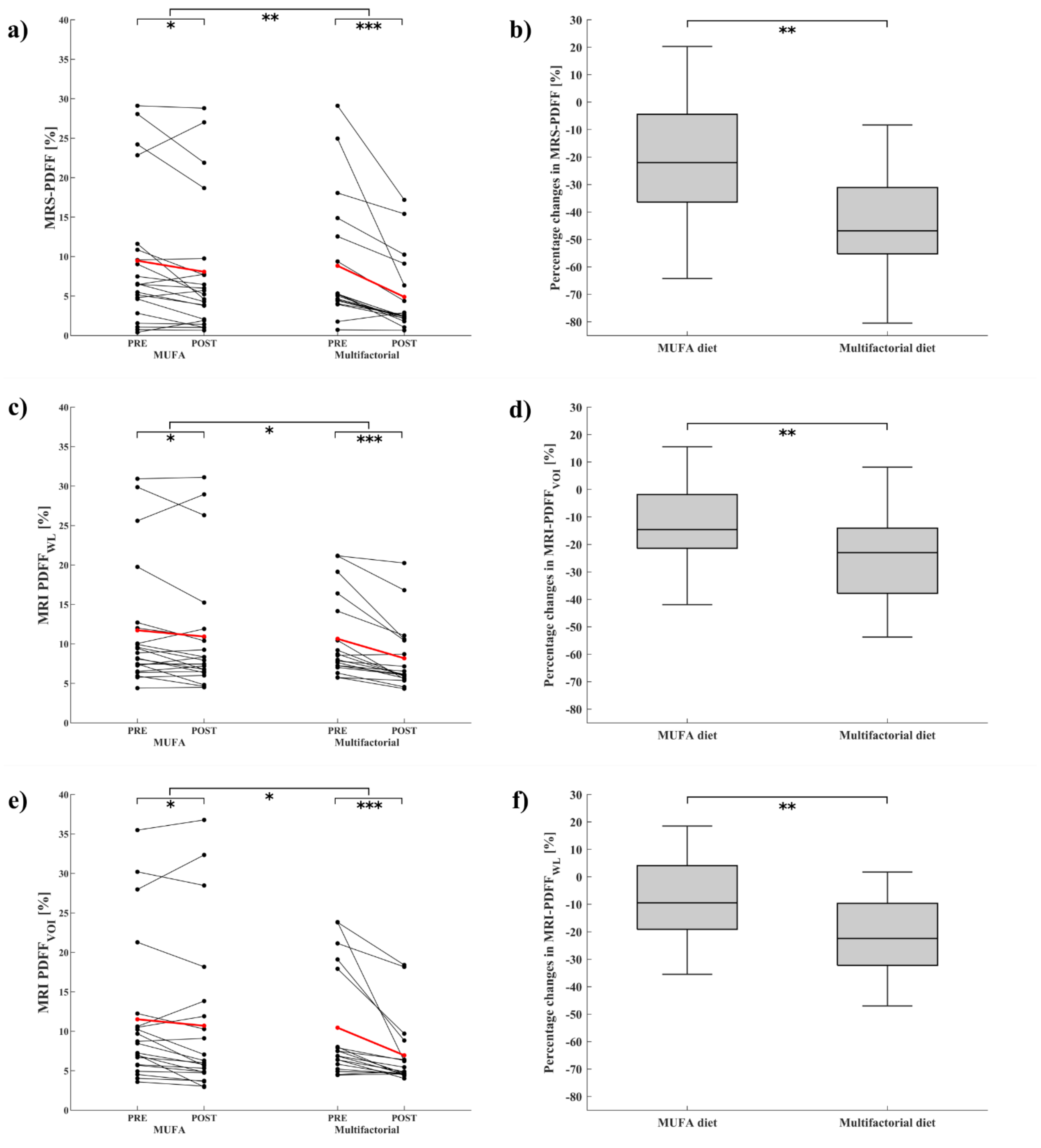

Figure S2. Absolute individual changes (left side) and percent changes (right side) in liver fat content after the 8-week intervention with MUFA diet or multifactorial diet, measured with MRS (a,b) and Dixon-MRI, with whole-liver-based (c,d) or VOI-based analysis (e,f). Asterisks indicates statistically significant differences (* = $P < 0.05$; ** = $P < 0.005$; *** = $P < 0.0005$). Abbreviations: MRI = Magnetic Resonance Imagnig; PDFF = Proton Density Fat Fraction; VOI = Volume Of Interest; WL = Whole Liver.
